# Antimicrobial resistance prevalence in *Escherichia coli* from German fattening pigs differs at subnational levels

**DOI:** 10.1128/aem.00878-25

**Published:** 2025-10-22

**Authors:** Maike Richter, Michael Weber, Timo Homeier-Bachmann, Carina Leitner, Susanne Amler, Carsten Heydel, Christa Ewers, Christian Berens, Christian Menge

**Affiliations:** 1 Friedrich-Loeffler-Institut, Institute of Molecular Pathogenesis 240210 , Jena, Germany; 2 Friedrich-Loeffler-Institut, Institute of Epidemiology 39023, Greifswald-Isle of Riems, Germany; 3 Justus Liebig University Giessen, Institute of Hygiene and Infectious Diseases of Animals 9175, Giessen, Germany; 4 Friedrich-Loeffler-Institut 39023 , Greifswald-Isle of Riems, Germany; Centers for Disease Control and Prevention, Atlanta, Georgia, USA

**Keywords:** antimicrobial resistance, surveillance, pigs, *Escherichia coli*, monitoring

## Abstract

The European Union aggregates surveillance data for antimicrobial resistance (AMR) in livestock at the member state level. However, data evaluated nationally do not allow regionalized risk assessment and reactions. In an attempt to estimate the magnitude and granularity of regional differences, we collected AMR data for *Escherichia coli* by sampling fecal matter using boot swabs from vehicles carrying fattening pigs to an abattoir with a cross-regional catchment area. One *E. coli* strain was isolated per animal transport and its susceptibility to 14 antimicrobials was determined by broth microdilution. The minimum inhibitory concentrations obtained served to group the isolates into wild type (without a resistance phenotype) and non-wild type (with a resistance phenotype) using epidemiological cut-off values. Isolates were classified based on the origin of the respective transport into different levels of regionalization, using administrative as well as artificial borders, to obtain subnational AMR prevalence estimates for comparisons. The resistance prevalence of the total data set (*n* = 992) matched well with German data from national AMR surveillance. At all regional levels assessed, we detected significant differences for different compounds, including one highly significant difference for the veterinary antibiotic enrofloxacin. The antibiotics sulfamethoxazole and tetracycline, with resistance prevalence spanning 27.6%–52.4% and 18.8%–43.2%, respectively, most frequently showed significant differences in resistance prevalence. Our findings demonstrate that rapid sampling of animal transporters with boot swabs at a central node in the production chain and subsequent assignment of the regional source of the animals can serve as an approach to unveil existing subnational AMR prevalence differences with reasonable effort.

**IMPORTANCE:**

Surveillance is a key element of the World Health Organization’s Global Action Plan on Antimicrobial Resistance (AMR). With livestock representing a potential source for AMR transmission, its inclusion in surveillance programs is indispensable. Governmental surveillance of livestock in the European Union only depicts nationally aggregated AMR data. However, obtaining AMR data at subnational levels, although possibly laborious, is essential to gain insights into local situations. These may differ depending on the region surveyed, but their knowledge is essential for targeted risk assessment and interventions. Our study aimed at collecting such data while simultaneously testing a time-saving sampling approach. Hence, we sampled animal transporters carrying fattening pigs to an abattoir with boot swabs and determined AMR prevalence for *Escherichia coli* strains isolated thereof. The significant subnational differences in AMR prevalence detected indicate that regionalized surveillance data improve and hone the AMR knowledge base. Furthermore, our sampling approach was applicable with reasonable effort.

## INTRODUCTION

The global presence of antimicrobial-resistant bacteria in livestock is a pressing issue, impacting animal health, welfare (1, 2), and the economy (3). The abundance of antimicrobial-resistant bacteria also threatens human health, due to bacteria with zoonotic potential (4) as well as commensals being a possible source for transmission of antimicrobial resistance (AMR) encoding determinants to bacteria pathogenic in humans (5–7). Observations indicating transmission of AMR genes and/or resistant bacteria between humans, animals, and the environment (8, 9) stress the need to address the AMR threat in a One Health Approach (9, 10). This is reflected in various action plans on AMR at both the global (11) and national (12) levels. In the European Union (EU), the Commission Implementing Decision 2020/1729/EU binds member states to monitor the AMR status of specific zoonotic and indicator bacteria along the food chain in common livestock species (13). They are obliged to report results for at least 170 isolates per year to the European Food Safety Authority (EFSA). EFSA publishes, together with the European Centre for Disease Prevention and Control, a detailed analysis of the annual AMR situation in humans, animals, and food (4). This harmonized AMR monitoring allows analysis and comparison at the member state level, but conclusions cannot be drawn at subnational levels. Indeed, the AMR situation might differ regionally, even within a country (14, 15), due to various influencing factors (16), such as farming management practices (17), external environmental effects (18), and different antimicrobial treatment preferences by veterinarians and/or farmers (19). Subnational estimates would represent a more suitable basis for the prioritization and strengthening of limited surveillance resources and, most importantly, for the application of targeted interventions (14, 20).

The sample matrix specified in the Commission Implementing Decision 2020/1729/EU for the origin of indicator commensal *Escherichia coli* isolates is cecal content, taken from one or more carcasses at the slaughterhouse. This selection aims to estimate the entry of the bacteria into the slaughterhouse (21), a sampling site where animals from many different areas congregate.

Taking samples from trucks carrying animals to a slaughterhouse could be an option to estimate AMR prevalence at the primary production level without the need to visit the originating farms, making comprehensive surveillance more feasible. Furthermore, sampling at the unloading ramp avoids entry into the inner areas of the slaughterhouse, where stricter hygiene regulations for food production apply. Using boot swabs to sample the transporters is an easy and quick approach to obtain specimens of fecal bacteria shed by the animal group during their transport. Boot swab sampling is routinely applied for monitoring *Salmonella enterica* subsp. *enterica* serovars Enteritidis and Typhimurium in broiler flocks in the EU (22) and, furthermore, has proven suitable for other bacteria and applications (23), particularly in the field of animal husbandry (24). For example, this method is recommended as a diagnostic tool for diarrhea in weaner pigs (25) and applied for the detection of extended-spectrum β-lactamases (ESBL)-, AmpC- (26), and carbapenemase-producing *E. coli* (27) on pig farms.

Therefore, our study aimed to obtain subnational prevalence data by using boot swab sampling on fattening pig transporters following their unloading at an abattoir as a measure to collect substantial numbers of samples, reflecting significant numbers of geographically spread-out pig holdings, with reasonable effort. We targeted *E. coli*, a commonly monitored indicator bacterium for AMR (28), and assessed differences in AMR prevalence on farms located in different regions of Germany.

## RESULTS

### *E. coli* isolates from fattening pigs and geographic sample distribution

Out of 1,022 transports sampled, 1,011 could be traced back to the originating municipality based on the truncated farm registration numbers provided for each transport. In the case of 172 transports, the fattening pigs were not delivered from a single farm but from 2 to 10 different farms (Table 1; Table S1). In contrast to “individual transports” with fattening pigs originating from a single farm, these were designated as “group transports.” Here, the fattening pigs most frequently (70%) came from only two different farms. Because the metadata only permitted resolution to the municipality level, “group transports” with pigs originating from the same municipality were also classified as “individual transports.” The geographic distances between the municipalities of origin from all other “group transports” varied greatly, ranging from being close to each other, even at the borders of two or three federal states, to being located up to 500 km apart. To enable a comparison and to classify the “group transports” properly, up to 153 transports were included at the administrative level used in the respective comparison group, while 19 transports, which could not be assigned to a common subnational administrative unit, were excluded from further analysis (Table S2). Eventually, analysis of the total data set was based on 992 transports with one *E. coli* isolate each.

**TABLE 1:** Composition of "group transports" (*n* = 172) with respect to number and estimated geographic location of the farms which supplied the fattening pigs on the respective transport

No. of farms sampled per transport	Total no. of transports	No. of “group transports” (*n* = 172) per lowest shared administrative unit			
		Municipality	County	Federal state	Country^*a*^
2	121	28	51	32	10
3	19	2	8	6	3
4	11	–^*b*^	2	7	2
5	11	–	3	7	1
6	3	–	–	1	2
7	4	–	1	3	–
8	1	–	1	–	–
10	2	–	–	1	1
Total	172	30	66	57	19

^*a*^ Estimated geographic locations of the farms from the “group transports” that belong to more than one federal state but are not (necessarily) associated with our geographical classification level of the regions comprising three to four federal states.

^*b*^ “–” indicates that there are no transports in the respective category.

**Fig 1:**
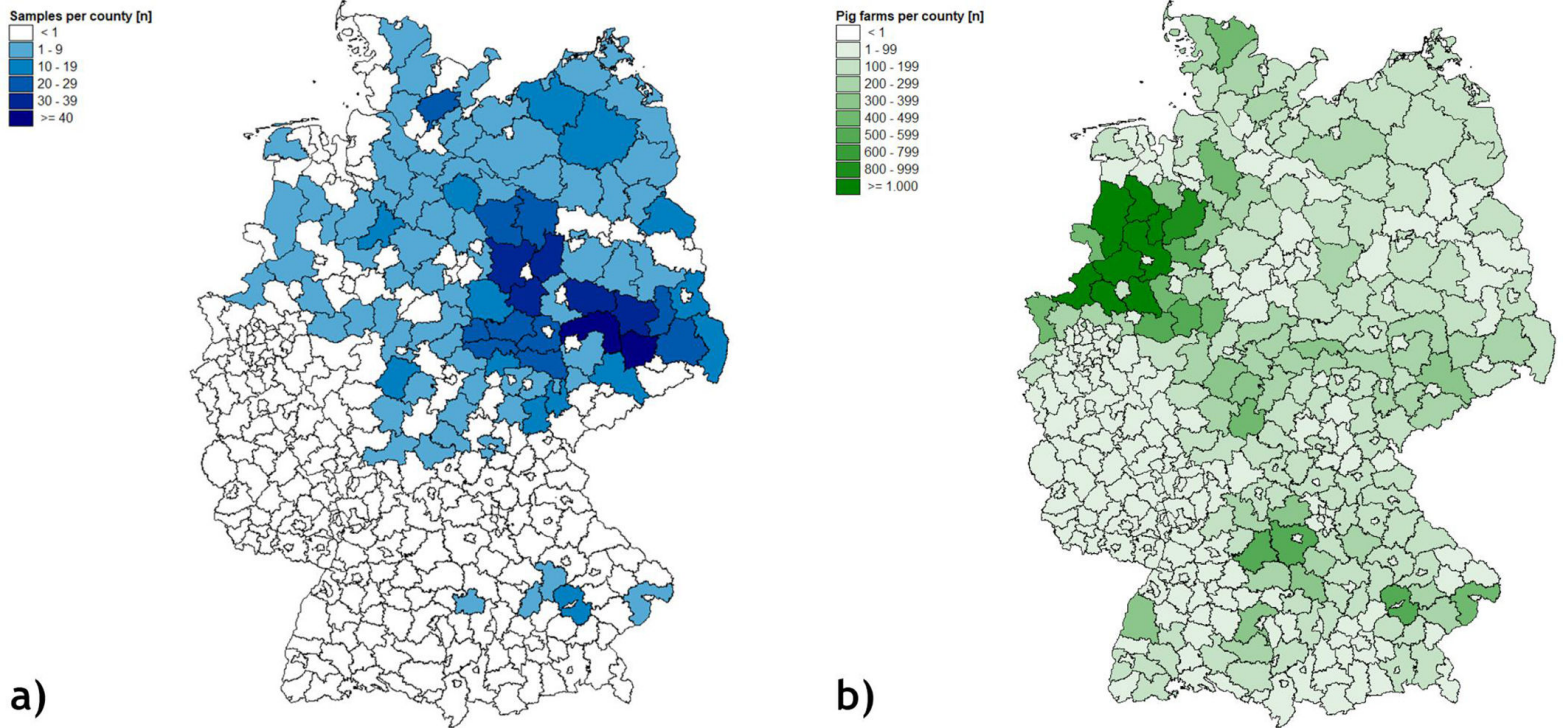
(**a and b**) Map of Germany depicting the numbers per county of (**a**) transports sampled in this study (*n* = 935) and (**b**) fattening pig farms in 2021 (https://www.hi-tier.de). Only “individual transports” and “group transports” with farms located in the same municipality or county were included.

The estimated locations of the farms, from which the fattening pigs were delivered to the slaughterhouse, were broadly distributed over the Northern half of Germany, with the majority located in the federal states of Saxony-Anhalt, Saxony, and Thuringia (Fig. 1a). Several farms were located in the north of Schleswig-Holstein, the west of North Rhine-Westphalia, and Southern Bavaria. Comparing the per-county density of our samples with the density of fattening pig farms per county in Germany reported for 2021 (Fig. 1b), sampling does not reflect farm density for the whole of Germany, because the northwestern area with the highest density of fattening pig farms is underrepresented.

### Antimicrobial susceptibility profiles of the *E. coli* isolates

**Fig 2:**
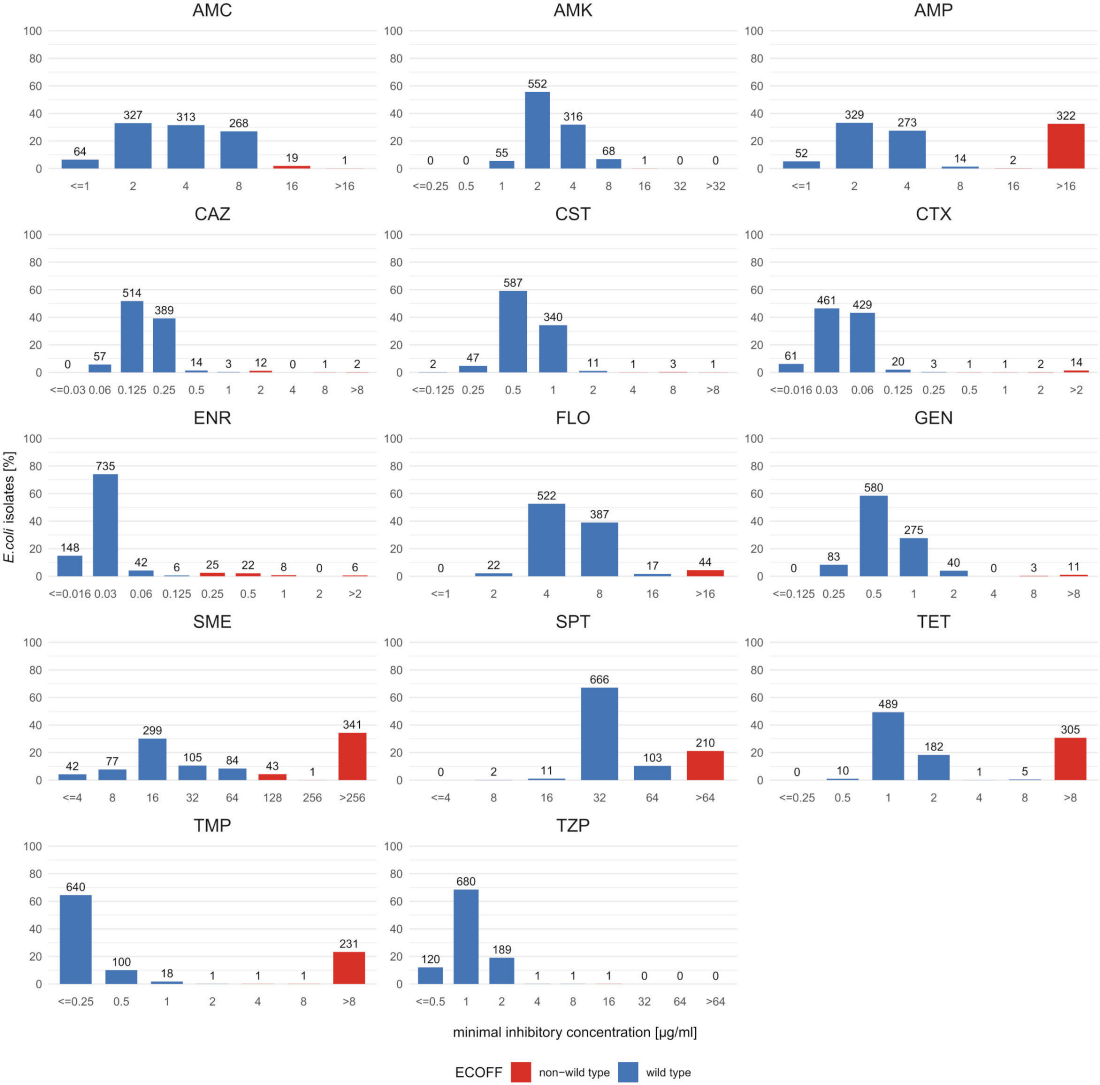
Relative and absolute distribution of the *E. coli* isolate (*n* = 992) MICs disaggregated for each antibiotic. AMC, amoxicillin/clavulanate; AMK, amikacin; AMP, ampicillin; CAZ, ceftazidime; CST, colistin; CTX, cefotaxime; ENR, enrofloxacin; FLO, florfenicol; GEN, gentamicin; SME, sulfamethoxazole; SPT, spectinomycin; TET, tetracycline; TMP, trimethoprim; TZP, piperacillin/tazobactam.

All isolates were subjected to antimicrobial susceptibility testing (AST) with a custom microtiter plate featuring 14 antibiotics and antibiotic/inhibitor combinations. These were chosen to (i) represent antibiotics relevant for human medicine (cefotaxime, ceftazidime, colistin, and piperacillin/tazobactam) (29) and licensed for veterinary use in pigs (all except amikacin, cefotaxime, ceftazidime, and piperacillin/tazobactam) (30), (ii) include antibiotics not present in current surveillance schemes (piperacillin/tazobactam and spectinomycin) (31, 32), and (iii) comprise antibiotics with published data indicative of varying levels of resistance prevalence concerning *E. coli* from fattening pigs (33–35). The MIC results from the AST are shown in Fig. 2 and in Table S3 and were interpreted according to epidemiological cut-off values (ECOFFs) from the European Committee on Antimicrobial Susceptibility Testing (EUCAST) as strains being either resistant or susceptible (Table 2; Table S3). Table 2 also displays the calculated MIC50/90 values, defined as the concentrations at which 50% and 90% of the tested bacterial population are inhibited in their growth. For the antibiotics and antibiotic/inhibitor combinations amikacin, amoxicillin/clavulanate, cefotaxime, ceftazidime, colistin, enrofloxacin, florfenicol, gentamicin, and piperacillin/tazobactam, the respective resistance prevalence was below 7%, whereas for the antibiotics ampicillin, spectinomycin, sulfamethoxazole, tetracycline, and trimethoprim, it was above 20%.

**TABLE 2:** Total numbers and resistance prevalence with corresponding 95% confidence intervals of *E. coli* strains^*a*^

Antibiotic	% resistant (*n*)	Confidence interval (95%)	MIC50 (µg/mL)	MIC90 (µg/mL)
Amikacin	0.10 (1)	0.02–0.57	2	4
Amoxicillin/clavulanate	2.02 (20)	1.31–3.09	4/2	8/4
Ampicillin	32.66 (324)	29.81–35.64	4	32
Cefotaxime	1.81 (18)	1.15–2.85	0.03125	0.0625
Ceftazidime	1.51 (15)	0.92–2.48	0.125	0.25
Colistin	0.50 (5)	0.22–1.17	0.5	1
Enrofloxacin	6.15 (61)	4.82–7.82	0.03125	0.0625
Florfenicol	4.44 (44)	3.32–5.90	4	8
Gentamicin	1.41 (14)	0.84–2.35	0.5	1
Piperacillin/tazobactam	0.10 (1)	0.02–0.57	1/4	2/4
Spectinomycin	21.17 (210)	18.74–23.82	32	128
Sulfamethoxazole	38.81 (385)	35.83–41.88	32	512
Tetracycline	30.75 (305)	27.95–33.69	1	16
Trimethoprim	23.49 (233)	20.96–26.23	0.25	16

^*a*^ MIC50 and MIC90 values of all *E. coli* strains (*n* = 992) are shown for each antibiotic tested.

The MIC distributions of the isolates are shown as bar charts (Fig. 2). As any breakpoint, irrespective of whether it is epidemiological or clinical, might change over time with ongoing research, ideally, the original MIC values should be supplied as a standard (36), enabling valid comparisons in future studies. For most antimicrobials and antimicrobial/inhibitor combinations, particularly those with a low prevalence of resistant isolates, the MICs were unimodally distributed with only a twofold difference in their MIC50 and MIC90 values. In contrast, the MIC distributions for ampicillin, sulfamethoxazole, tetracycline, and trimethoprim, which had a high resistance prevalence, were bimodal and showed three- to sixfold higher MIC90 values compared to the MIC50 values. Concerning trimethoprim, more than 60% of the isolates had a MIC less than or equal to 0.25 µg/mL, whereas nearly a quarter displayed a MIC higher than 8 µg/mL. The MIC distribution of spectinomycin, with a resistance prevalence of 21%, did not show a clear uni- or bimodal distribution, but rather lacked a distinct separation between isolates classified as susceptible or resistant and displayed only a fourfold difference between the MIC50/MIC90 values.

**Fig 3:**
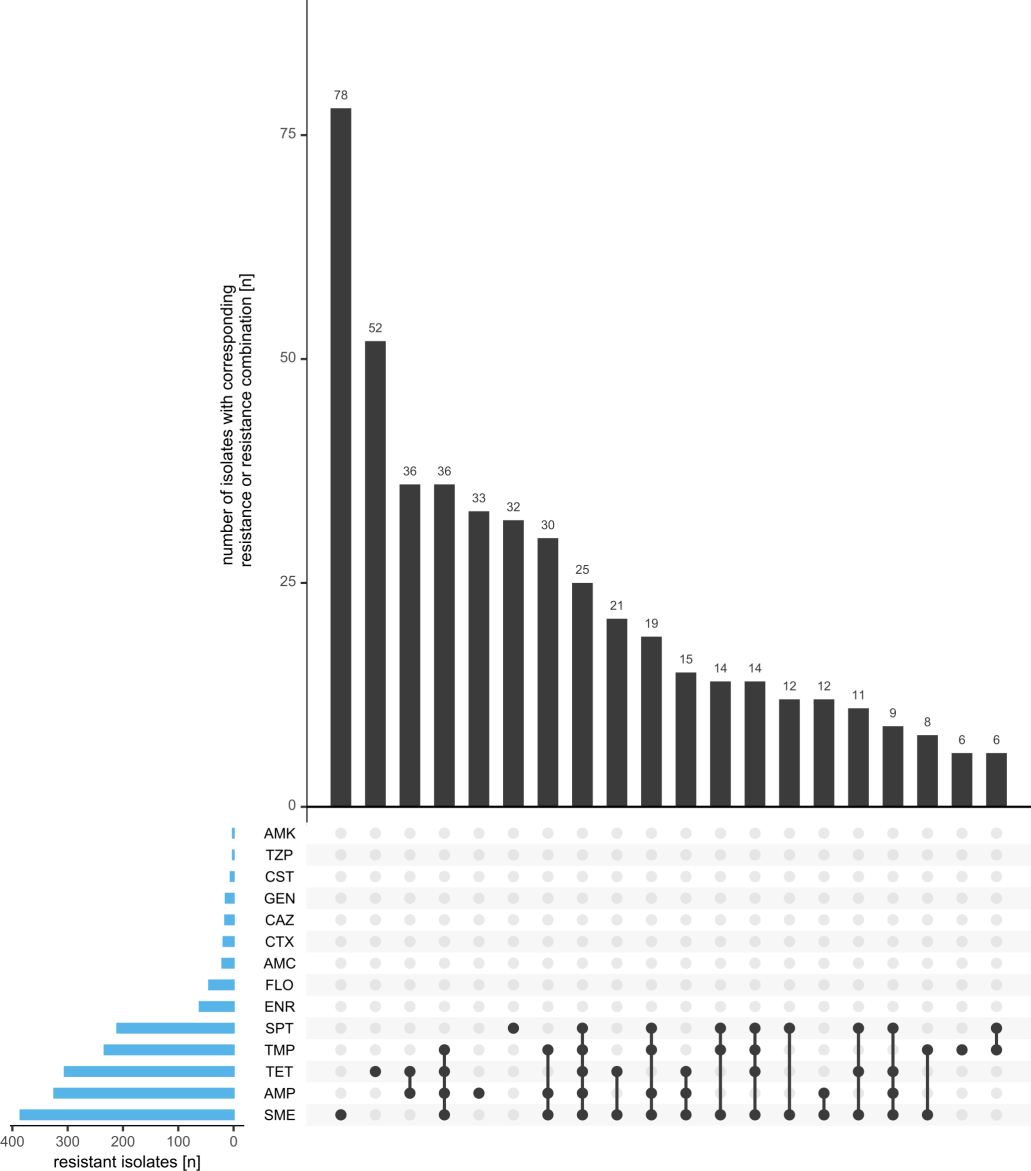
Distribution of *E. coli* isolates displaying the 20 most prevalent resistance profiles (excluding complete susceptibility [CS]). In the combination matrix, each distinct phenotypic profile is depicted. A black dot stands for resistance to an antibiotic compound. The vertical black bar chart represents the total number of isolates with a specific phenotypic profile, whereas the horizontal blue bar chart displays the number of isolates resistant to each antibiotic compound. AMC, amoxicillin/clavulanate; AMK, amikacin; AMP, ampicillin; CAZ, ceftazidime; CST, colistin; CTX, cefotaxime; ENR, enrofloxacin; FLO, florfenicol; GEN, gentamicin; SME, sulfamethoxazole; SPT, spectinomycin; TET, tetracycline; TMP, trimethoprim; TZP, piperacillin/tazobactam.

**Fig 4:**
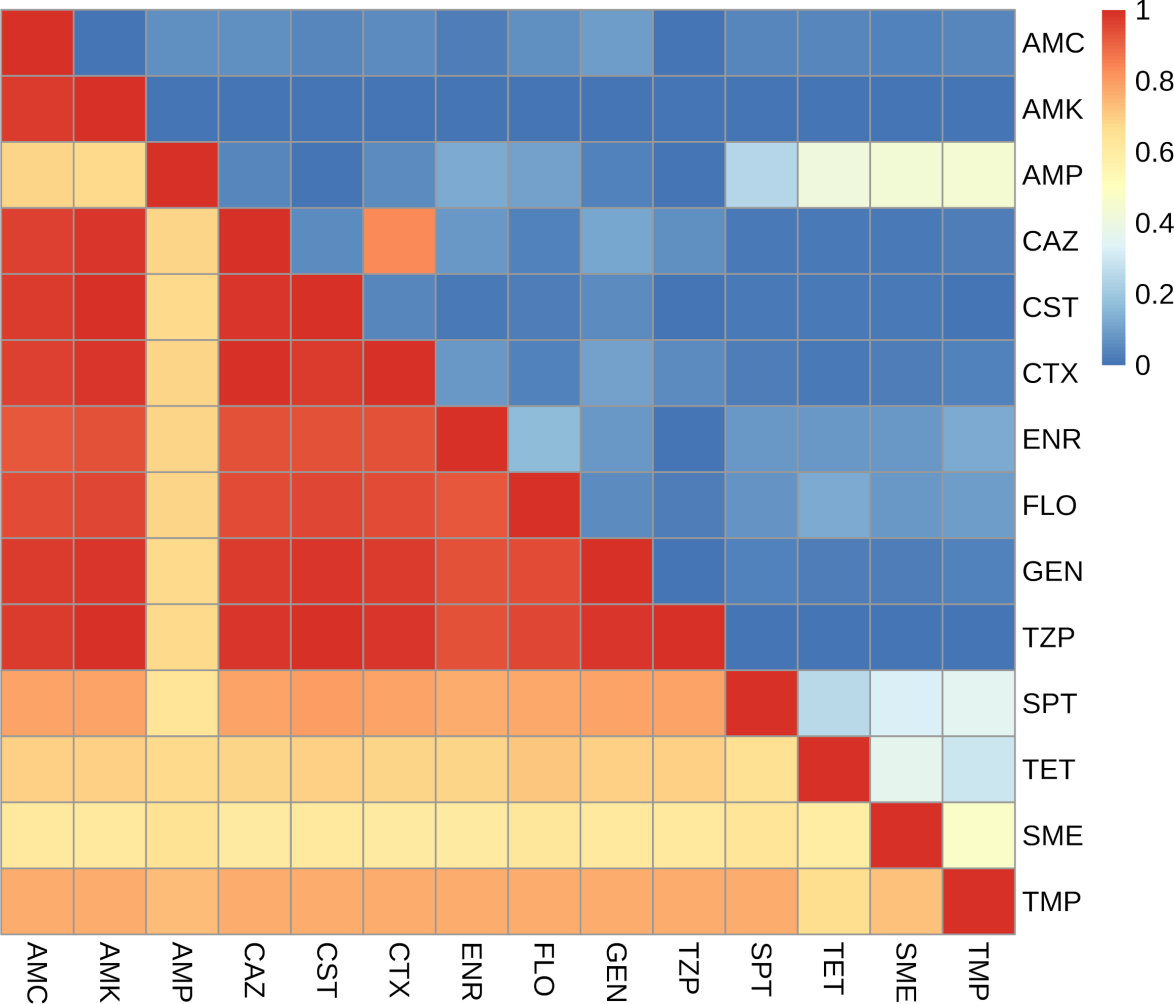
Heatmap of Jaccard indices showing the probability of an *E. coli* isolate being resistant (upper triangular)/susceptible (lower triangular) to both antimicrobial agents when it is resistant (upper triangular)/susceptible (lower triangular) to one of those agents. AMC, amoxicillin/clavulanate; AMK, amikacin; AMP, ampicillin; CAZ, ceftazidime; CST, colistin; CTX, cefotaxime; ENR, enrofloxacin; FLO, florfenicol; GEN, gentamicin; SME, sulfamethoxazole; SPT, spectinomycin; TET, tetracycline; TMP, trimethoprim; TZP, piperacillin/tazobactam.

By far, the most frequent pattern detected among the 992 *E. coli* strains tested was “complete susceptibility” (CS; 37.8%) to all antibiotics and antibiotic/inhibitor combinations on the panel. For the remaining strains, Fig. 3 shows the 20 most frequent profiles out of all 109 profiles (Table S4), with sulfamethoxazole (7.9%), tetracycline (5.2%), ampicillin (3.3%), and spectinomycin (3.2%) being the most common single-antibiotic resistances. Approximately half of the resistance profiles (*n* = 52) were found in just a single isolate. Figure 4 depicts the association between a susceptible or resistant phenotype for each pair of antimicrobial agents. Susceptibility index (SI) values for associations between susceptible phenotypes, ranging from 0.60 (sulfamethoxazole and tetracycline) to 1.0 (amikacin and piperacillin/tazobactam), are overall higher than the resistance index (RI) values for associations between resistant phenotypes. For the latter, several combinations did not show any association. The highest RI value (0.83) was obtained for cefotaxime and ceftazidime, both third-generation cephalosporins. Also, high values were observed for ampicillin in combination with sulfamethoxazole (0.44), tetracycline (0.41), or trimethoprim (0.44), as well as for sulfamethoxazole and trimethoprim (0.47). Figure 3 shows that several isolates were resistant to two or more antibiotics and antibiotic/inhibitor combinations. Here, the five most frequent resistance profiles were ampicillin + tetracycline (36 isolates; RI = 0.41), ampicillin + tetracycline + sulfamethoxazole + trimethoprim (36 isolates; RI = 0.16), ampicillin + sulfamethoxazole + trimethoprim (30 isolates; RI = 0.30), ampicillin + tetracycline + spectinomycin + sulfamethoxazole + trimethoprim (25 isolates; RI = 0.08), and tetracycline + sulfamethoxazole (21 isolates; RI = 0.36).

**Fig 5:**
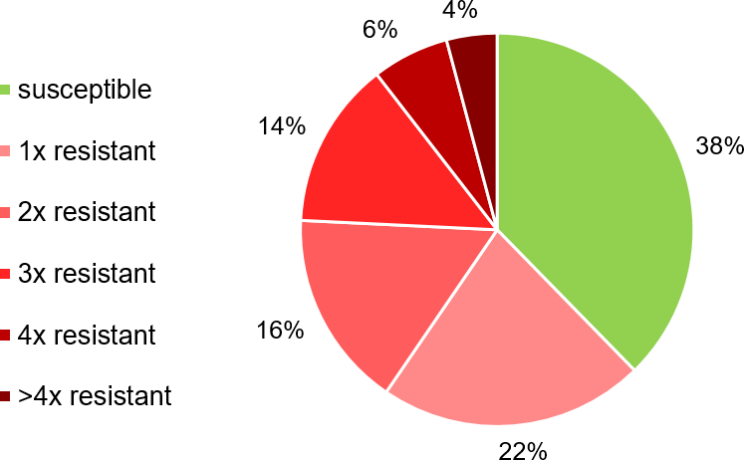
Proportion of *E. coli* isolates (*n* = 992) showing a susceptible or a resistant phenotype for up to more than four antimicrobial classes assessed in this study.

Multidrug resistance (MDR) was present in 24% of the isolates (Fig. 5). Two of the *E. coli* strains even showed resistance toward antimicrobials from seven of the antibiotic classes present on the panel (Table S4).

Resistance to ceftazidime and/or cefotaxime was detected in 18 *E. coli* isolates. They were obtained from samples of farms located in five federal states without obvious clustering. A phenotypic confirmatory test for the ESBL phenotype revealed that 15 of the 18 *E. coli* isolates were ESBL producers. All isolates with an ESBL phenotype were also resistant to ampicillin but not to the antibiotic/inhibitor combination piperacillin/tazobactam. One of the isolates showed a resistant phenotype against the antibiotic/inhibitor combination amoxicillin/clavulanate.

### Regional profiling of resistance prevalence

**Fig 6:**
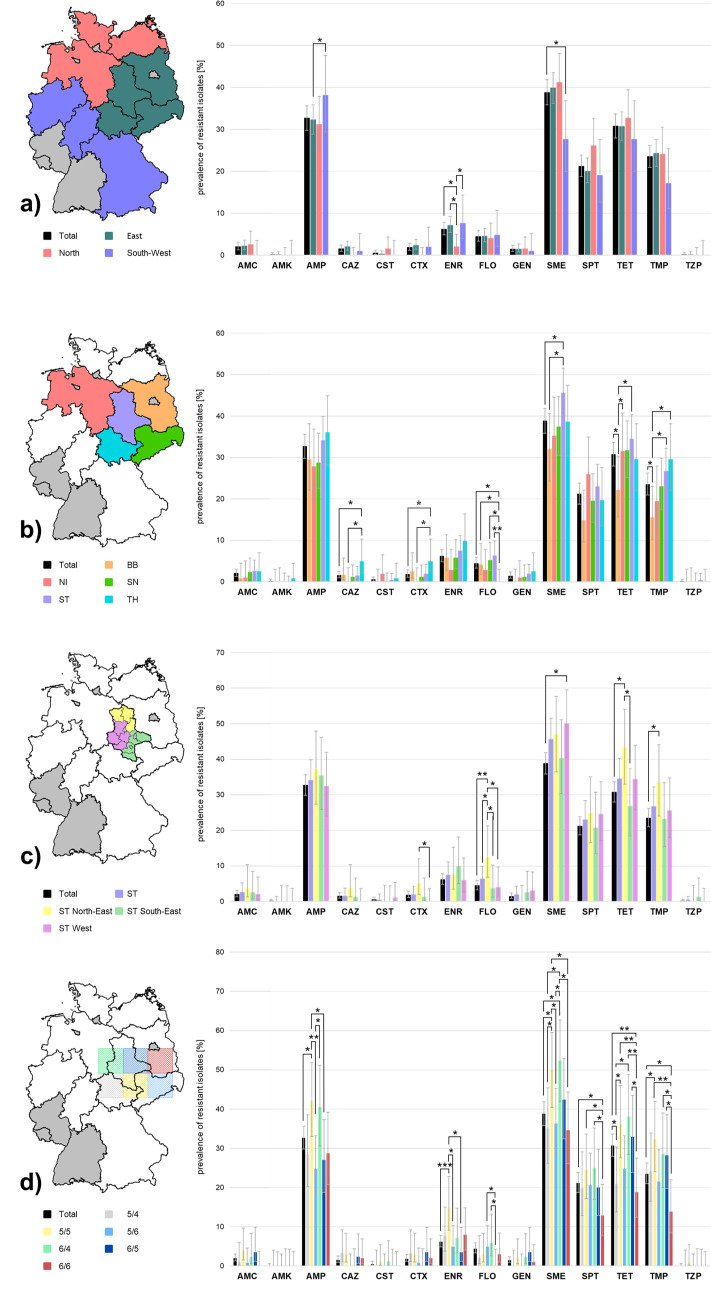
(**a–d**) Percentage of resistant *E. coli* isolates for (**a**) regions combining several German federal states, (**b**) federal states, (**c**) sub-state regions combining several counties in Saxony-Anhalt, and (**d**) grid squares (numbers indicate the position of the grid as depicted in Fig. S2). No samples were obtained from the federal states colored gray. Isolates from the sectors colored white were not considered in the respective prevalence calculations. AMC, amoxicillin/clavulanate; AMK, amikacin; AMP, ampicillin; CAZ, ceftazidime; CST, colistin; CTX, cefotaxime; ENR, enrofloxacin; FLO, florfenicol; GEN, gentamicin; SME, sulfamethoxazole; SPT, spectinomycin; TET, tetracycline; TMP, trimethoprim; TZP, piperacillin/tazobactam; BB, Brandenburg; NI, Lower Saxony; SN, Saxony; ST, Saxony-Anhalt; TH, Thuringia; *P ≤ 0.05, **P ≤ 0.01, and ***P ≤ 0.001 (Fisher‘s exact test/*χ*^2^ goodness-of-fit test); error bars represent the 95% confidence intervals.

To obtain antibiotic resistance and MDR prevalence at subnational levels for comparison, isolates were classified based on the municipal localization of their originating farms. Analysis started with larger organizational and administrative units, proceeding to smaller units or an artificial grid square system (Table S1). The results are presented as bar charts (Fig. 6; Figure S1), each in comparison to our aggregated total prevalence data as reference. Exact values of subnational prevalence estimates of each geographical classification are listed in Tables S5 to S8.

At first, the sampling area was divided into three regions, termed “East,” “North,” and “South-West,” by grouping three to four federal states into a region (Fig. 6a; Fig. S1a). At this level, weakly significant differences in prevalence were detected between the regions for several antibiotics. Ampicillin resistance was more prevalent in the “South-West” region compared to the “East” region. Conversely, resistance to sulfamethoxazole was less prevalent in the “South-West” region compared to the overall data set. The “North” region had a lower prevalence of enrofloxacin resistance relative to the other regions and the total data set.

Differences in strain resistance prevalence were also detected at the level of individual federal states—both between each other and with respect to the total data set (Fig. 6b; Fig. S1b). Only federal states with a sufficient number of samples to allow determination of a resistance prevalence were included in this analysis (*n* = 108–270; Table S6). The federal state of Thuringia displayed a significantly lower resistance prevalence for florfenicol when compared to Saxony-Anhalt (*P* = 0.002). All other differences were weakly significant. This was the case for the low resistance prevalence calculated for Thuringia for florfenicol in comparison to two other federal states and the total data set (*P* = 0.01–0.03). In contrast, Thuringia had a higher resistance prevalence with respect to Lower Saxony and the entire data set for the third-generation cephalosporins ceftazidime and cefotaxime. On the other hand, Saxony-Anhalt showed a higher resistance prevalence for sulfamethoxazole when compared with our overall data or with Brandenburg. Lastly, Brandenburg had a lower resistance prevalence than the total data set and several federal states with respect to the antibiotics trimethoprim and tetracycline. Also, in Brandenburg, the level of CS was higher in comparison to any other federal state (excluding Saxony) and the entire data set. In Saxony-Anhalt, more multidrug-resistant *E. coli* isolates were identified than in Brandenburg (Fig. S1b).

Assessing differences within a federal state was possible for Saxony-Anhalt (ST) from which 265 *E. coli* isolates were obtained. This number of samples made it feasible to divide the federal state into three sub-state regions, each combining three or four counties, referred to as “ST North-East,” “ST South-East,” and “ST West” (Fig. 6c; Fig. S1c). In the “ST North-East” region, a significantly higher prevalence of resistance to florfenicol was detected (*P* = 0.003) compared to the overall prevalence estimate. In addition, weakly significant higher resistance prevalence was determined (i) for tetracycline and trimethoprim compared to the entire data set, (ii) to the resistance prevalence determined for the entire federal state of Saxony-Anhalt for florfenicol, and (iii) also to one or both other regions for cefotaxime, florfenicol, and tetracycline. Also, the “ST West” region showed a weakly significant higher prevalence of resistance to sulfamethoxazole relative to the overall data set. A significantly higher ratio of multidrug-resistant *E. coli* isolates was detected for the “ST North-East” region compared with Saxony-Anhalt and the total data set (Fig. S1c).

Finally, a different approach that is independent of administrative geographic borders and commonly utilized in epizootic disease control (37) was applied. In this approach, a grid with constant edge lengths is spread over a region or an entire state, and the livestock farms contained within each grid square are identified. The objective is to determine the farm and animal densities objectively, based exclusively on spatial extent. This enables the definition of specific disease control measures for individual grid squares. In this study, the *E. coli* strains obtained were to be grouped at the subnational level in order to objectify the analysis of the data. Grid squares numbered from “1/0” to “9/10” and had a fixed side length of 100 km each (Fig. S2). Farms sampled were present in 32 grid squares, and six of these grid squares contained sufficient sample sizes (*n* = 84–121; Table S8) to allow prevalence calculation and comparisons (Fig. 6d; Fig. S1d). Also, samples originating from municipalities divided by the boundaries of grids were included and assigned to the grid in which the Kartenexplorer software located their potential origin. With this categorization, the individual grid squares yielded more and higher significant differences among themselves and compared to our overall prevalence estimate than the other classifications. The grid square “5/5” showed a higher resistance prevalence in comparison to the total data set concerning nearly every antibiotic tested, except for amikacin and florfenicol, including weakly significant (ampicillin, sulfamethoxazole, and trimethoprim) and highly significant (enrofloxacin) differences. Furthermore, also weakly significant (ampicillin, *P* = 0.01, “5/6”; tetracycline, *P* = 0.004, “6/6”; trimethoprim, *P* = 0.003, “6/6”) differences compared to other grid squares were determined. Also, the MDR levels determined from the grid square “5/5” were weakly significantly higher in comparison to other grid squares (“6/6”; “5/6”) and to the entire data set analyzed. Simultaneously, CS was observed less frequently in this sample set as well as for the grid square “6/4” with differences reaching significance toward other grid squares and the total data set (“6/4”; *P* = 0.03). In general, the grid square “6/6” displayed lower resistance prevalence compared to the entire data set with respect to nearly every antibiotic, except for cefotaxime, ceftazidime, and enrofloxacin. These differences were weakly significant for the antibiotics spectinomycin and trimethoprim and even significant for tetracycline (*P* = 0.009). Moreover, weakly significant and significant (tetracycline, *P* = 0.004, “6/4”) differences were also identified compared to other grid squares. Consistent with these results, CS levels of the grid square “6/6” were weakly significantly higher than in our overall data and significantly higher compared to the grid squares “6/4” and “5/5.”

## DISCUSSION

### Transport sampling at a slaughterhouse as an alternative approach to surveillance

The decision to sample animal transporters at a cross-regional slaughterhouse was aimed at minimizing sampling efforts while still obtaining a sufficient number of samples from its catchment area for subnational AMR prevalence determination. In a similar approach in Italy, pooled fecal samples were taken from the truck floor immediately after the fattening pigs had been unloaded at a large abattoir to check for the presence of hepatitis E virus, an emerging foodborne pathogen (38). In our study, boot swabs were used to sample the transporters because they are a user-friendly, quick, and established approach to obtain an appropriate sample of the bacteria shed by an animal group (24). German Zoonoses Monitoring in 2017 and 2019 analyzed both fecal samples from farms as well as cecal samples from slaughterhouses for the animal group of fattening pigs in the respective years. When comparing the reported resistance rates for both matrices assessed in the same year with each other, we determined that they did not differ significantly, except for gentamicin in 2019 (*P* = 0.009) (39, 40).

Operations at a cross-regional slaughterhouse are strictly coordinated (41), and any disturbance of the processes can be very disruptive. Thus, truck sampling had to be done in a timely manner and, for safety reasons, after the pigs had been unloaded. At this time point, only one level of the truck was accessible, which can affect the representativeness of the boot swab sample for the entire group of pigs when transported on several floors of the truck or trailer. Animal transportation can play a role in the spread of infectious diseases (41, 42), which raises the question of cross-contamination between transport trips in our study. Cold water power-washing of trucks at Irish slaughterhouses, which had previously transported fattening pigs, was not always sufficient to reduce the bacterial load to prevent possible cross-contamination, particularly with enterobacteria such as *Salmonella* (43). In a recent study investigating bacterial and viral fecal contamination at four Italian slaughterhouses, no *Salmonella* was isolated from four trucks after washing (44). Based on the fact that cleaning and disinfection procedures of trucks before leaving the abattoir are mandatory in the EU (45), we regarded the risk of cross-contamination from previous transports conducted by the vehicles in our survey as minor.

When comparing the sample distribution from our survey with the density of fattening pig farms from Germany in 2021 (Fig. 1), our sampling did not fully represent the entire German pig fattening industry. This would have required contacting and visiting slaughterhouses located in Western and Southern Germany. Estimation of the national prevalence in Germany was not intended, as such data are available from the Zoonoses Monitoring (21) and EFSA reports (4). Rather, this study aimed at introducing a novel sampling approach for unveiling geographic differences with reasonable effort. In total, we included samples from 98 distinct counties, roughly a quarter (24.4%) of the 401 German counties and urban municipalities. The median and mean numbers of fattening pig farms in the sampled counties were roughly twice as high as the national average, indicating that regions with higher pig fattening intensity were more likely to be sampled. Yet, the latter is additionally influenced by the size of the individual fattening pig farm. Importantly, our sample collection covered a geographic area large enough to calculate subnational prevalence for geographic regions with structural differences in animal husbandry (46), as well as for several federal states. This enabled comparisons not only with the total data set but also among administrative units at different organizational levels. Such an analysis has not been performed to date to this extent.

### Aggregated AMR estimates in the context of antimicrobial categorization by the European Medicines Agency and relative to existing data

Based on the 992 *E. coli* isolates, we identified resistance prevalence above 20% and up to nearly 40% for the antibiotics ampicillin, spectinomycin, sulfamethoxazole, tetracycline, and trimethoprim. According to the Antimicrobial Advice *Ad Hoc* Expert Group categorization of antibiotics for prudent and responsible use in animals, these are all classified in the category D “Prudence,” which is intended for first-line treatment (47). The high prevalence of resistance to these antibiotics is not unexpected considering that compounds belonging to these antibiotic classes have been used extensively in both veterinary and human medicine (48, 49). All five antibiotics are classified as “highly important antimicrobials” from the “WHO list of medically important antimicrobials” (29) for human medicine. Therefore, the results appear to be less critical in terms of consumer protection, although potential cross-resistance to determinants mediating resistance to critically important antimicrobials for human medicine should not be neglected. Nevertheless, their importance for the treatment of bacterial infections in food-producing animals has to be kept in mind as they are all classified as “veterinary critically important antimicrobials” by the World Organisation of Animal Health (30). If these antibiotics could not be used further as a therapy option, this would impact animal welfare (1, 2).

The resistance prevalence for amikacin (0.0%), ampicillin (28.9%), cefotaxime (0.0%), ceftazidime (0.0%), colistin (0.0%), gentamicin (2.6%), tetracycline (32.7%), and trimethoprim (23.7%) for *E. coli* isolates obtained from cecal samples of slaughtered pigs sampled in 2021 as part of the German Zoonoses Monitoring was similar compared to the corresponding estimates in our study (32). Only for sulfamethoxazole (29.5%) did we detect a significantly higher (*P* = 0.008) resistance prevalence of 38.8%. Consequently, we consider our data set to be appropriate for evaluating potential subnational prevalence differences.

### Few resistance profiles predominate in the pig population

As stated in Ruddat et al., the diversity of a sample set can be assessed by examining the number of distinct resistance profiles in relation to the number of isolates analyzed (50). With 14 different antimicrobials and antimicrobial combinations in our panel and a binary outcome variable for the *E. coli* isolates as being either resistant or susceptible, theoretically 2¹⁴ = 16,384 different resistance profiles would be possible. Not all of these will be realized, since cross-resistance of a resistance determinant to several antibiotics present in the panel will eliminate a subset of these combinations. Nevertheless, the 109 different resistance profiles among the 992 isolates contained in our sample set indicate a relatively limited diversity. A similar relationship between the number of resistance profiles and the sample size was found for *S*. Typhimurium isolates originating from Lower Saxony in Germany and associated with sporadic salmonellosis cases (51), whereas for *E. coli* strains isolated from composite fecal samples of fattening pigs from Canada, the profile-to-sample ratio was considerably lower (52) and, thus, even more restricted. This limited resistance profile diversity is also mirrored by (i) the 10 most prevalent resistance profiles, which contain 59% of the isolates with at least one phenotypic resistance (362/617) and (ii) the 38% completely susceptible *E. coli* isolates.

The SI values for our *E. coli* isolates were much higher than the RI values, reflecting that the majority of the isolates were susceptible to most of the antibiotics tested. The degree of CS to all antimicrobials tested in the EFSA panel for commensal *E. coli* isolated from the cecal content of fattening pigs in Germany was higher (49.5%), yet the results of our study are similar regarding the median prevalence (38.3%) of all 28 EU member states (53). In contrast, a higher median MDR prevalence (31.2%) was reported for all member states but not for Germany (22.6%). The latter was comparable to the degree of MDR (24.1%) determined for the *E. coli* strains from our survey (53).

The highest values for the two-dimensional RI of different antibiotic classes were 0.41 for the combination of ampicillin and tetracycline, which was also one of the most frequent resistance profiles, and 0.47 for the combination of sulfamethoxazole and trimethoprim. The linkage between both antibiotic combinations is plausible because ampicillin and tetracycline belong to the two antibiotic classes with the highest quantities dispensed in Germany for livestock (54), and trimethoprim and sulfonamides are part of many combined antibiotic compounds licensed for livestock species. Moreover, genes coding for resistance against the latter two antibiotics are frequently found jointly on mobile genetic elements, leading to their co-selection (52). It stands out that RIs were generally high for combinations of antibiotics, with overall high detected resistance rates, increasing the probability that those would occur simultaneously. The second most frequent multidimensional resistance profile (ampicillin + sulfamethoxazole + trimethoprim) possessed an RI of 0.3, which was also the most frequent MDR profile among commensal *E. coli* isolates from pigs in Europe (53). A combination of the antibiotics ampicillin-tetracycline-trimethoprim/sulfamethoxazole was the most frequent resistance profile found in the German national resistance monitoring of animal pathogens for pigs of all age groups (31).

However, the previously mentioned RI values are not high enough to conclude that there is a meaningful association, with the exception of the combination of ceftazidime and cefotaxime, with the highest RI value (0.83). This result was to be expected as resistance against both third-generation cephalosporin antibiotics is mediated through ESBL and AmpC enzymes, imparting cross-resistance to many structurally similar β-lactam antibiotics (55). Consequently, all 15 *E. coli* isolates with an ESBL phenotype also displayed resistance against the β-lactam ampicillin. Quantifying the association of resistance phenotypes—which can be due to genetically linked resistance mechanisms, cross-resistance, the simultaneous use of those antibiotics, or co-selection caused by exposure to biocides (antiseptics, disinfectants, and preservatives) and heavy metals (56) such as copper and zinc frequently used as growth promoters—can be used to monitor dynamics in resistance patterns and hypotheses arising from this can initiate genomic research (50). In fact, whole-genome sequencing will be needed to determine the nature of the associated resistance determinants and their genetic linkage.

### Regionalization reveals subnational prevalence differences

We were able to detect subnational differences regarding the levels of CS and MDR in the *E. coli* isolates. As such differences are also observed between participating states of the EFSA monitoring, this was not unexpected. In these reports, even major differences were revealed for both MDR (8.2% in Finland to 78.8% in Spain) and CS (6.5% in Spain to 77.7% in Finland) ratios, with trends toward increasing levels of CS from south to north and east to west in Europe.

To estimate and compare prevalence data at subnational levels and with the total prevalence data, we applied different regionalization approaches to our data. The data protection restrictions allowed farm localization only to the municipality level. In addition, the metadata of the corresponding transports were only available after sampling, preventing sensible decision-making regarding which transports to sample. Thus, “group transports” were only considered when their common administrative level was included in the analysis. The general aim was to make the best use of the data for checking to what extent and down to which level regional differences could possibly be detected. First, we divided our sampling area into the three regions “East,” “North,” and “South-West,” each including three to four federal states, aiming for a balanced sample number as best possible in each region. Applying this regionalization approach revealed a few weakly significant prevalence differences. A proposal to divide Germany into five distinct regions “Middle,” “Northwest,” “East,” “South,” and “Upper Rhine,” based on their agricultural structures concerning livestock farming for pigs, cattle, and laying hens, was made by Merle et al. (46). Their aim was to identify representative districts per region which could serve as a proxy to be analyzed in future surveys on livestock populations. This regionalization partly overlaps with our segmentation and would require sampling at one or more additional slaughterhouses. Hering et al. detected risk factors affecting farm management and hygienic aspects for cefotaxime-resistant *E. coli* in fattening pig farms from those representative districts (57). Although they were able to isolate ESBL-suspected *E. coli* with different frequencies from farms in the agricultural regions, they could not statistically confirm the region as a risk factor.

An obvious choice is to make use of the existing borders of federal states for a more granular regionalization. Using administrative borders for a regionalization approach is reasonable when taking potential official actions or regulations into account that could be implemented based on surveillance results. In this survey, the two federal states of Saxony-Anhalt and Thuringia exhibited significantly higher AMR prevalence for several antibiotics, except for florfenicol, compared to the other federal states analyzed and the total estimate. These results fit well with increased antibiotic dispensing quantities for veterinary medicine in Germany in 2021 for the south of Saxony-Anhalt. However, such comparisons must be made with caution as actual reports assign antibiotic use geographically to postal codes which do not correspond to federal states or counties (https://www.bvl.bund.de/SharedDocs/Pressemitteilungen/05_tierarzneimittel/2022/2022_PM_Abgabemengen_Antibiotika_Tiermedizin.html, last accessed on 20 April 2025). Yet, in 2021, antibiotic quantities were still recorded as the amount sold by pharmaceutical companies to veterinary practices, so it is not known where and when exactly the antibiotics were applied. Furthermore, no allocation to different animal species was possible because antibiotics are frequently authorized for use in more than a single animal species. Based on the EU regulation 2019/6 on veterinary medicinal products, the quantities of antibiotics dispensed to, used in, or prescribed for animals must now be systematically recorded for each animal species individually. This obligatory documentation is implemented incrementally, adding different groups of animal species every 3 years. In 2023, cattle, pigs, chickens, and turkeys were designated as the animal species for which data recording is mandatory at the initial implementation stage. This will allow more detailed analysis of the connections between antimicrobial use and resistance data in the future.

The next level of regionalization involved a more detailed analysis of resistance prevalence in the federal state of Saxony-Anhalt from which we had obtained the most samples. Here, classification into three regions, named “ST North-East,” “ST South-East,” and “ST West,” each combining up to three counties, was adopted. At this level, the “ST North-East” region stands out with up to significantly higher resistance prevalence for several antibiotics. Such higher AMR prevalence in geographical hotspots can provide an incentive for further investigation or for targeted interventions.

Although convenient, a classification based solely on administrative borders, which can differ, e.g., largely in size, farm and animal density, might not always be advisable. We therefore decided to alternatively apply a grid square-based regionalization approach, which has been utilized in animal health control settings (37). With this approach, significant prevalence differences were found with two grid squares having either a higher (“5/5”) or lower (“6/6”) AMR prevalence. As the area of the grid “6/6” comprises approximately the southern area of the federal state of Brandenburg, the results are in line with the lower AMR prevalence in Brandenburg as already mentioned. Mulchandani et al. also applied the concept of geographically dividing countries into pixels for modeling AMR predictions based on existing official surveillance data supplemented with point prevalence surveys, albeit at a smaller scale with a surface area of 100 km² instead of the 10,000 km² used here (14, 58). With this methodology, they predicted geographic hotspots with higher AMR prevalence within a country. Reasons for these differences might be multiple and need to be resolved to implement targeted interventions. An obvious reason would be the prescription habits of local veterinarians, which can be obtained, e.g., by means of a questionnaire and assigned to the corresponding farms, which might correlate with hotspots. However, catchment areas of practicing veterinarians neglect administrative boundaries as well, and their prescribing preferences can be an influential factor for AMR prevalence (19).

### Conclusion

In summary, our approach of using boot swabs to sample livestock transport vehicles at an abattoir proved effective for collecting a large number of specimens with reasonable effort. Given that our accumulated prevalence data correlate very well with data from the national Zoonoses monitoring, along with the advantages of sampling an entire animal group rather than individual carcasses at a limited number of locations, our sampling strategy might serve as a superior alternative. Applying the different regionalization concepts to our data allowed us to detect differences in the MDR/CS ratios and AMR prevalence, which turned out to be significant in several cases. Additional metadata, missing in our survey, would improve the analysis of factors contributing to these differences, as the geographic region is only a surrogate for many influencing factors. Still, our findings highlight the demand for knowledge of the subnational AMR prevalence situation. The substantial sample sizes needed for this cannot be neglected. Alternative surveillance strategies requiring less samples might facilitate implementation of regionalized prevalence studies (59–61).

## MATERIALS AND METHODS

### Slaughterhouse sampling

Sampling was performed at a cross-regional slaughterhouse in Central Germany on 26 days during 7 consecutive weeks (July 2021 to September 2021). The samples (*n* = 1,022) were taken from trucks at the unloading ramp, right after the fattening pigs had left the loading area of the vehicle. For sampling, single boot swabs were premoistened with buffered peptone water and packed in sterile plastic sample bags (Romerlabs, Butzbach, Germany). To prevent cross-contamination between the truck and slaughterhouse environment, boots were prepared with plastic boot covers that separated the boot from the swab. The so-prepared boots were only put on at the edge between the vehicle ramp and the loading area of the truck or trailer. The person doing the sampling walked the full length of one trailer level and back, with special focus on areas with much slurry and feces. Subsequently, the swab was carefully pulled off the plastic boot cover and placed back in its original sterile sample bag. Processed sample bags were kept in polystyrene boxes with cool packs until sampling had been completed and during the transport back to the laboratory. For the next sampling, boots were prepared with a fresh plastic cover and a new swab.

For each transport sampled, the corresponding registration number(s) of the originating farm(s) were recorded truncated by omitting the last three digits, following the data protection agreement with the slaughterhouse operating company. The shortened number allowed to trace the origin of the animal group sampled back to the municipality level (Table S1) but not to the farm itself.

### Sample processing

The sample bags were stored overnight at 4°C. The next day, 100 mL sterile buffered peptone water (Carl Roth, Karlsruhe, Germany) was added to the sample bag containing the boot swab and palpated manually for 1 minute (23). A 10 mL aliquot was removed and incubated overnight (18–22 h, 200 rpm) at 37°C. After incubation, an aliquot of the enriched suspension was supplemented with 25% glycerol in buffered peptone water (62) for further storage as a 10% glycerol suspension at −80°C.

### Isolation of *E. coli* strains

For *E. coli* strain isolation, the enriched suspensions were rapidly thawed at 50°C for 2–3 min (62). One inoculation loop (10 µL) was streaked out on MacConkey agar (sifin diagnostics, Berlin, Germany), and plates were incubated at 37°C for 18–22 h. One colony with typical coliform morphology was picked, sub-cultured on MacConkey and Endo agar plates (Sigma-Aldrich Chemie GmbH, Taufkirchen, Germany), and analyzed by Matrix Assisted Laser Desorption/Ionization Time of Flight using a score of ≥2.0 for species identification (63) until one *E. coli* strain had been identified in each sample. The isolates were stored with 75% glycerol in buffered peptone water at −80°C.

### Antimicrobial susceptibility testing

Each *E. coli* strain was analyzed for its antibiotic susceptibility profile via broth microdilution using a customized microtiter plate containing 14 antimicrobials and antimicrobial/inhibitor combinations (Table S9), based on the MICRONAUT AST system (MERLIN Diagnostika, Bornheim-Hersel, Germany). The concentrations of the antimicrobials were chosen to include clinical and epidemiological breakpoints and the quality control ranges of the reference strains selected. The *E. coli* isolates were streaked out onto blood agar (sifin diagnostics), and single colonies were picked to prepare suspensions corresponding to 0.5 McFarland units with a 0.9% NaCl solution (Carl Roth, Karlsruhe, Germany) and transferred to cation-adjusted Mueller-Hinton-Bouillon (Sigma-Aldrich Chemie B.V., The Netherlands). The microtiter plates were then inoculated with this bacterial suspension following the manufacturer’s instructions (MERLIN Diagnostika, Bornheim-Hersel, Germany) and incubated for 18–20 h at 37°C. After incubation, automated turbidity reading and interpretation with the use of pre-defined breakpoints was carried out using a microplate reader (Tecan, Crailsheim, Germany) and MCN6 software (MERLIN Diagnostika), except for the antibiotics trimethoprim and sulfamethoxazole, which were analyzed visually, because antagonists in the medium may lead to slight growth of the isolate despite susceptibility, which impedes an automated evaluation (64). The *E. coli* reference strains ATCC 25922 and NCTC 13846 served as quality control strains. In order to obtain MIC values for all strain/drug combinations to calculate with, the following was defined in case that (i) a strain was already inhibited by the lowest test concentration of the antibiotic tested or (ii) the highest antibiotic concentration of the panel was not able to inhibit a strain: for (i) the MIC value was set to the lowest antibiotic concentration and for (ii) the MIC value was set to the next antibiotic concentration in a twofold dilution series following the highest concentration tested (51). Applying the ECOFFs from EUCAST (https://mic.eucast.org/search/, last accessed on 3 August 2023) for *E. coli*, the isolates were differentiated according to their respective MIC value into non-wild type strains and wild-type strains (Table S3). For the antibiotic combination amoxicillin/clavulanic acid, the tentative ECOFF was used. Because no ECOFF was available from EUCAST for the antimicrobial sulfamethoxazole, the ECOFF proposed in the Commission Implementing Decision 2020/1729/EU was used (Table S9). When *E. coli* isolates were categorized as non-wild type concerning the antimicrobials cefotaxime and/or ceftazidime, a phenotypic confirmatory test for the expression of an ESBL-phenotype was performed using a VITEK 2 compact system (bioMerieux). Testing was performed using software version 9.02 and the AST-N428 and AST-XN24 cards, according to the manufacturer’s instructions.

### Terminology applied here

According to EUCAST, because non-wild-type bacterial strains are defined as having “phenotypically detectable acquired resistance mechanisms” (65) in contrast to wild-type bacterial strains, the former are correctly termed “microbiologically” resistant (4). For convenience of reading, however, they are referred to as “resistant” throughout this manuscript, and the bacterial strains classified as wild type are designated “susceptible.”

An *E. coli* isolate was classified as multidrug resistant when it was resistant to at least one member in each of three or more antibiotic classes (4) as defined in the “Categorisation of antibiotics in the European Union” (47). CS (4) was defined as the absence of phenotypic resistance to all substances present in the panel.

The Jaccard index, a measure of set similarity, was used to quantify the probability that an *E. coli* isolate is resistant or susceptible to both antimicrobial agents, given that it is resistant or susceptible to one of them. This metric is hereafter referred to as the resistance/susceptibility index as described in Ruddat et al. (50).

### Data processing and statistical analysis

The municipalities of origin of the pig transports, derived from the registration numbers, were mapped in a 100 km × 100 km grid over the entire Federal Republic of Germany. In addition to the AST results of the individual isolates and the total number of farms of origin, the individual grid cells were additionally enriched with the number of pig farms from the HIT database (https://www.hi-tier.de/).

Estimated true prevalence, including the respective confidence intervals and assuming a sensitivity and specificity of 100%, was determined using the Epitools Epidemiological Calculators (https://epitools.ausvet.com.au/trueprevalence) with default settings and Wilson interval approach to calculate the 95% CI for apparent prevalence (66). The required sample size to detect the expected AMR prevalence was determined using EpiRechner, version 0.5 (https://shiny.fli.de/ife-apps/EpiRechner/), defining a precision of 10% and a statistical certainty of 95% applying different population sizes (https://www.hi-tier.de/) and expected prevalence values based on national AMR monitoring data (31, 32) and published literature (33, 35). For a graphical representation of the distribution of the samples, maps were prepared using Kartenexplorer, version 2.2 (https://kartenexplorer.fli.de/).

Statistical analyses were performed using the R statistical environment (version 4.2.0). Sample intersections of resistance to the 14 substances were visualized using an Upset plot, which was generated with the UpSetR package (version 1.4.0). Jaccard indices were computed using the vegdist function in the vegan package (version 2.6.6) and visualized with pheatmap (version 1.0.12). The bar plots illustrating the MIC distributions and the resistance profiles were generated using ggplot2 (version 3.4.4).

Fisher’s exact test was calculated using the fisher.test() function to compare AMR, MDR, and CS prevalence between the members of each of the different geographical classifications (region, federal state, sub-state region in Saxony-Anhalt, grid). Significance at the 5%-levels was defined as: “weakly significant” (*P* ≥ 0.01 to *P* ≤ 0.05), “significant” (*P* ≥ 0.001 to *P* < 0.01), and “highly significant” (*P* < 0.001), respectively. To compare resistance prevalence calculated for the abovementioned different geographical units to the overall prevalence of the total data set, the *χ*^2^ goodness-of-fit test was applied using the function chisq.test(). If the expected frequencies (*n* < 5) were too low, the exact *P*-value was estimated through repeated sampling (Monte Carlo simulation) with 10,000 repetitions. No adjustment for multiple testing was applied.

## SUPPLEMENTAL MATERIAL

File S1 aem.00878-25-s0001.xlsx Tables S1 and S3 to S9.

File S2 aem.00878-25-s0002.pdf Table S2 and Fig. S1 and S2.

ASM does not own the copyrights to Supplemental Material that may be linked to, or accessed through, an article. The authors have granted ASM a non-exclusive, world-wide license to publish the Supplemental Material files. Please contact the corresponding author directly for reuse.
