# Supplementary material for: Antimicrobial resistance prevalence in Escherichia coli from German fattening pigs differs at subnational levels
Source: Appl Environ Microbiol. 2025 Oct 22;91(11):e00878-25. doi: 10.1128/aem.00878-25 (PMC12628832; doi:10.1128/aem.00878-25)
Supplement: File S2 — Table S2 and Fig. S1 and S2. [file aem.00878-25-s0002.pdf]

Table S2: Total numbers of *Escherichia coli* isolates from individual and group transports included on each level in the analysis of the geographical classification group

Geographical classification	No. of *E. coli* isolates included from	
	Individual transports	Group transports
Region	839	153
Federal state*	701	95
Sub-state regions of ST	255	10
Grid*	555	29

*Only federal states and grids with a sample number sufficient to allow determination of a resistance prevalence were included in this analysis. ST= Saxony-Anhalt

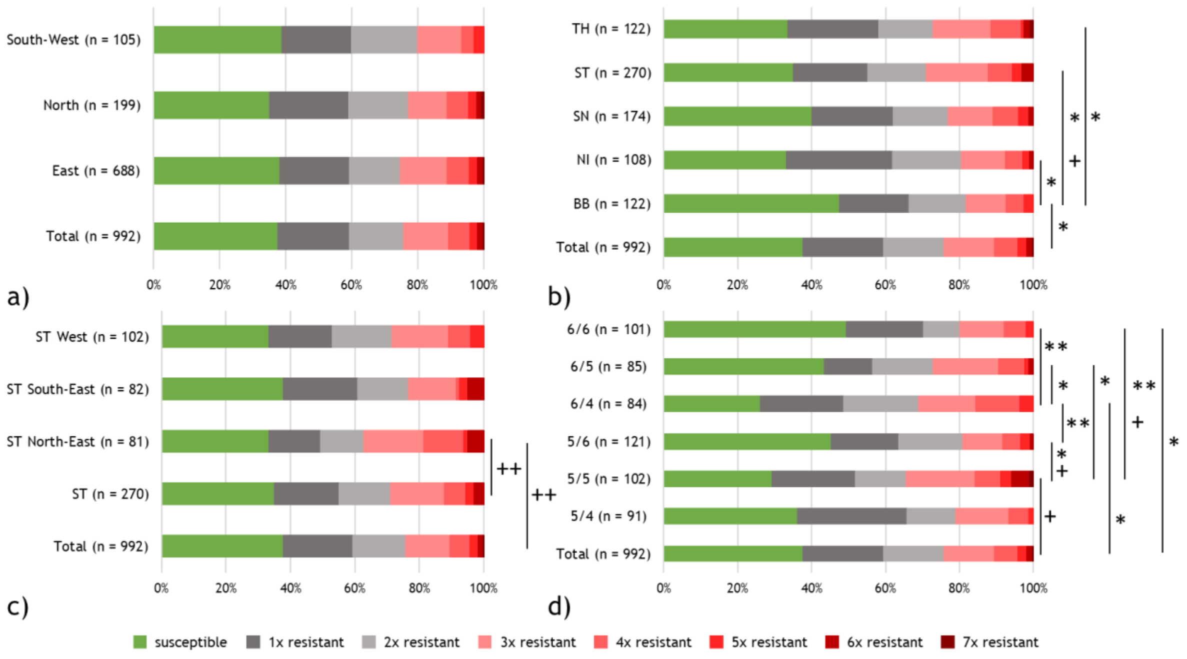

Figure S1: Percentages of *Escherichia coli* isolates showing either complete susceptibility or resistance towards an increasing number of antibiotic classes in a) regions combining several German federal states, b) federal states, c) sub-state regions combining several counties in Saxony-Anhalt and d) grid squares (numbers indicate the position of the grid as depicted in figure S2). Asterisks/crosses representing the levels of significance for differences concerning ratios of CS/MDR. BB, Brandenburg; NI, Lower Saxony; SN, Saxony; ST, Saxony-Anhalt; TH, Thuringia; */+ $p \leq 0.05$, **/++ $p \leq 0.01$ (Fisher's exact test/chi-squared goodness-of-fit test).

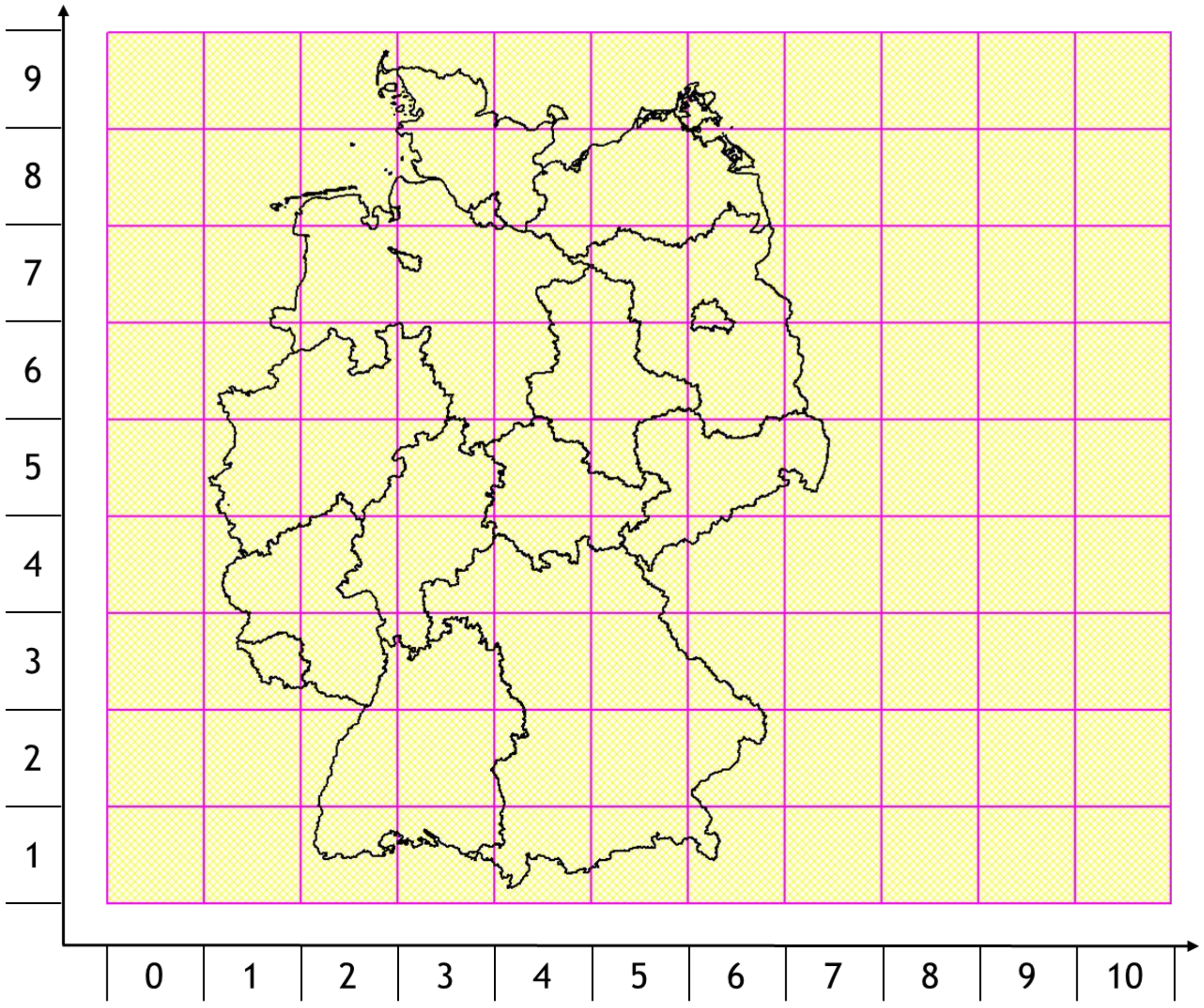

Figure S2: Grid of 100 km x 100 km squares “1/0” to “9/10” generated over the map of Germany. The y-axis represents the first and the x-axis the second numeral.
